# Correlations between shear wave elastography and mammographic findings in invasive breast cancer

**DOI:** 10.1186/bcr2949

**Published:** 2011-11-04

**Authors:** MJ Budak, K Thomson, P Whelehan, S Vinnicombe, A Evans

**Affiliations:** 1 Dundee Cancer Centre, Ninewells Hospital and Medical School, Dundee, UK

## Introduction

Shear wave elastography is a new ultrasound technique which shows promise in the differential diagnosis of solid breast masses. This study compares the mean tumour stiffness with mammographic findings and mammographic density in women with invasive breast cancer.

## Methods

Mammographic morphological features and BIRADS density assessment of the contralateral breast were documented in 96 consecutive patients with operable invasive breast cancer who had undergone shear wave elastography. Mammographic assessment was performed blinded to the elastography and pathological findings. Mean stiffness values of individual tumours were classified as either above or below the average mean stiffness of the cancers. Chi-squared tests for trend were used to compare mean stiffness with mammographic features and density.

## Results

There was no statistically significant difference in the mean stiffness of tumours according to their mammographic features. Mean tumoural stiffness increased with breast density (average mean stiffness 126 kPa, 132 kPa, 154 kPa, and 179 kPa for BIRADS densities 1 to 4, respectively), with a statistically significant trend of stiffness being above average for a cancer in a denser breast. No statistically significant correlation was shown between tumour size or grade (factors known to be associated with increased stiffness) and mammographic density.

## Conclusion

Tissue stiffness of breast cancers is greater in women with mammographically denser breasts. Assuming elastographic stiffness reflects characteristics of stroma and tumour, our findings suggest that tumour-stroma interactions may vary with mammographic density.

